# Recurrent Candida parapsilosis Complex Endocarditis in a Patient With a Mechanical Mitral Prosthetic Valve Managed Conservatively in a Resource-Limited Setting: A Case Report

**DOI:** 10.7759/cureus.108143

**Published:** 2026-05-02

**Authors:** Walter A Villeda, José Arturo Portillo, Katlheen Nolasco Ferrera, Rene J Santos

**Affiliations:** 1 Infectious Diseases, Honduran Social Security Institute, San Pedro Sula, HND; 2 Cardiology, Honduran Social Security Institute, San Pedro Sula, HND; 3 Internal Medicine, Universidad Nacional Autónoma de Honduras, San Pedro Sula, HND

**Keywords:** blood culture, candida parapsilosis infection, infective endocarditis, prosthetic heart valve, rheumatic heart disease

## Abstract

Infective endocarditis is an infection of the endocardium and cardiac valves. Fungal etiologies are rare, accounting for 1-3% of cases, and affect prosthetic valves in 0.1% of cases. We present a case of recurrent fungal endocarditis, caused by *Candida parapsilosis *in a patient with a mechanical mitral prosthetic valve successfully managed with medical therapy alone in a resource-limited setting. This report highlights the diagnostic and therapeutic challenges of fungal endocarditis and demonstrates that under appropriate clinical conditions, such as the absence of congestive heart failure, heart block, thrombotic events, and close monitoring with appropriate antifungal therapy, favorable outcomes may be achieved even in the absence of surgical intervention.

## Introduction

Infective endocarditis is a disease characterized by infection of the endocardium and cardiac valves. *Staphylococcus aureus* remains the most frequent causative agent; however, fungi of the *Candida *genus have become increasingly clinically important, particularly among patients with extensive healthcare exposure and invasive procedures [1,2].

Fungal endocarditis accounts for approximately 1-3% of all infective endocarditis cases but is associated with mortality rates exceeding 50%. This infection is strongly linked to prosthetic valves, prior cardiac surgery, use of central venous catheters, and immunosuppressive states. Among *Candida* species, *Candida parapsilosis* represents a particular clinical challenge due to its ability to form biofilms on prosthetic material, facilitating persistent infection and antifungal resistance [3-5]. Combined antifungal therapy and surgical intervention are the standard treatments for this condition, considering whether patients meet the criteria for surgical management and whether surgery is a feasible option [1].

The following case report aims to describe the clinical course of a patient with a history of mechanical mitral and aortic valve replacement, with recurrent fungal endocarditis due to *Candida parapsilosis*, which was successfully treated using conservative management with combined antifungal therapy, without surgical intervention. This case contributes to the growing literature on conservative management of fungal endocarditis in resource-limited contexts, underscoring the importance of individualized therapeutic decisions and close clinical monitoring. This report adheres to the CARE (CAse REports) guidelines [6].

## Case presentation

A 41-year-old man, non-immunosuppressant and non-drug user, with a history of rheumatic heart disease complicated by mitral and aortic valvulopathy, underwent mechanical valve replacement in both positions in October 2022.

During his first hospital admission, the patient presented with intermittent fever, demonstrated with temperature fluctuation between 100.4°F and 102.2°F lasting three months, accompanied by arthralgias, myalgias, and violaceous macular lesions on the fingertips. Serial blood cultures yielded *Candida parapsilosis* complex in multiple samples, identified utilizing the BD Phoenix™ M50 system (Becton, Dickinson and Company (BD), USA) (Table 1). Antifungal susceptibility with minimum inhibitory concentration (MIC) values was not performed due to the unavailability of technology, such as matrix-assisted laser desorption ionization-time of flight (MALDI-TOF) and VITEK 2, within the hospital or the city. Due to the absence of infectious disease consultation services at the institution and economic limitations that precluded further diagnostic evaluation, empirical fluconazole therapy was initiated at a dose of 400 mg intravenously every 24 hours for 14 days, resulting in clinical improvement and negative follow-up blood cultures. The electrocardiogram showed no evidence of heart block or congestive heart failure (Figure 1). Echocardiography was not performed at that time; this episode was managed as candidemia. A summary of the patient’s clinical course is presented in Table 2.

**Table 1:** Timeline of blood cultures

Date and tests	Result
November 7, 2023	
Bacterial blood culture	Negative
Fungal blood culture	Positive
Identified organism	*Candida parapsilosis* complex
April 8, 2024	
Bacterial blood culture	Negative
Fungal blood culture	Positive
Identified organism	*Candida parapsilosis *complex
April 20, 2024	
Bacterial blood culture	Positive
Fungal blood culture	Negative
Identified organism	Staphylococcus lugdunensis
April 28, 2024	
Bacterial blood culture	Negative
Fungal blood culture	Negative
Identified organism	None
October 18, 2024	
Bacterial blood culture	Positive
Fungal blood culture	Positive
Identified organism	*Staphylococcus capitis*; *Candida parapsilosis* complex
November 21, 2024	
Bacterial blood culture	Negative
Fungal blood culture	Negative
Identified organism	None

**Figure 1:**
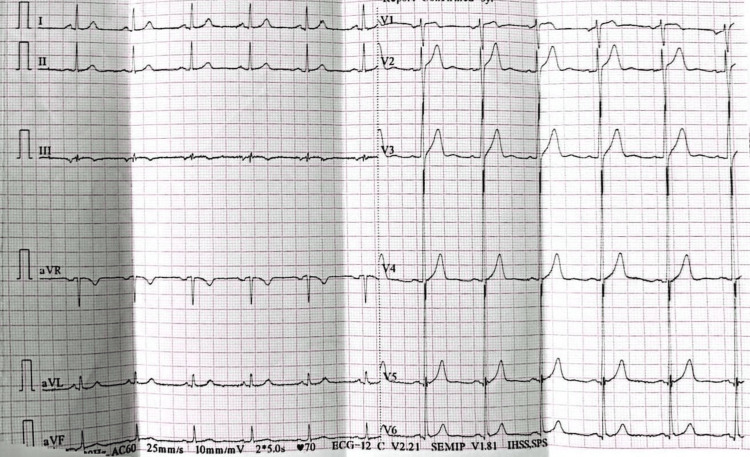
12-lead electrocardiogram during the first hospital admission Standardized, sinus rhythm, normal cardiac axis, no atrioventricular blocks, prominent and symmetrical T waves in V2-V5 consistent with early repolarization, no signs of ischemia

**Table 2:** Timeline of events

Date	Event
2015	Diagnosis of rheumatic heart disease. Managed as an inpatient by the cardiology department.
January 2022	Identification of unspecified mitral and aortic valvulopathy.
October 2022	Surgical replacement of the mitral and aortic valves with mechanical prostheses.
October 2023	First hospital admission due to candidemia. Empirical treatment with fluconazole was initiated. No infectious disease consultation was conducted, as the service was unavailable at the hospital at that time.
Early 2024	The patient remained afebrile, followed as an outpatient under oral antifungal therapy (fluconazole PO).
April 2024	Second hospital admission due to fungal endocarditis. Blood cultures positive for *Candida parapsilosis*; diagnosis confirmed by transesophageal echocardiography (TEE). Managed by the infectious disease service with caspofungin and intravenous fluconazole.
Early November 2024	Third hospital admission due to recurrent fungal endocarditis. Blood cultures were found positive for *C. parapsilosis *again, sample taken on October 18, 2024, during an outpatient consultation. Antifungal therapy was administered according to international guidelines (amphotericin B deoxycholate and intravenous caspofungin). The patient declined surgical intervention.
Late November 2024	Favorable clinical course; the patient was afebrile with negative blood cultures.
December 2024	The patient was discharged three weeks after a negative culture in November under oral antifungal therapy (fluconazole PO) and scheduled outpatient consultation follow-up.
2025	The patient remains afebrile, with negative blood cultures and no evidence of complications or recurrences.

The patient was readmitted with persistent fever and new violaceous maculopapular lesions on the fingers; no signs or symptoms of chest congestion were present. Blood cultures were again positive for *Candida parapsilosis *complex, demonstrating recurrence of fungemia as detailed in Table 1, and a holosystolic murmur was detected at the mitral focus. Transthoracic and transesophageal echocardiography revealed a 14.4 mm vegetation attached to the mitral prosthesis (Figure 2). The aortic valve had a normally functioning double-disc mechanical valve prosthesis, the tricuspid valve had moderate regurgitation at 2.74 m/sec, and the pulmonary valve had a normal structure. There was no abscess, valvular perforation, aneurysm, or dehiscence present.

**Figure 2:**
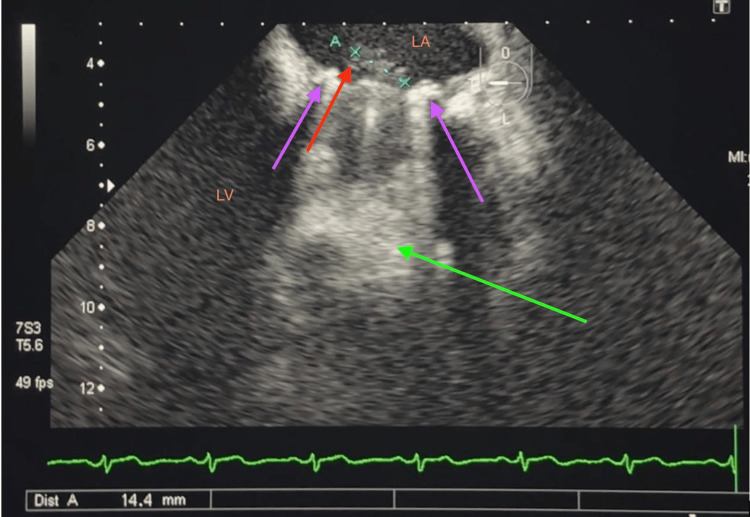
Mitral valve vegetation visualized through transesophageal echocardiography (TEE) during the first recurrence Red arrow: vegetation measuring 14.4 mm in length. Green arrow: posterior acoustic shadowing. Purple arrow: mitral valve. LA: left atrium, LV: left ventricle

The modified Duke criteria were used to help diagnose infective endocarditis, given that the patient had one major criterion and three minor criteria. Likewise, the patient had risk factors, such as a prosthetic heart valve and rheumatic heart disease [2]. 

A multidisciplinary team recommended surgical intervention; however, the patient declined surgery due to financial constraints. Medical management was initiated with intravenous caspofungin (50 mg/day) and fluconazole (1200 mg/day) for six weeks. Amphotericin B was unavailable, and the full recommended dose of caspofungin could not be administered due to institutional shortages.

Despite these limitations, the patient showed clinical improvement, with resolution of fever and reduction of vegetation size on follow-up echocardiography. He was discharged with negative blood cultures and chronic suppressive oral fluconazole therapy.

Six months later, the patient experienced another relapse while receiving fluconazole prophylaxis, which was not suspended, presenting with fever and recurrent cutaneous lesions. Blood cultures again confirmed *Candida parapsilosis* complex, as detailed in Table 1. Laboratory findings during this second recurrence demonstrated elevated inflammatory markers with preserved renal function, which are summarized in Table 3.

**Table 3:** Laboratory findings during the second recurrence of hospitalization

Laboratory test	Result	Reference range
Red blood cell line		
Red blood cells	5.14 × 10⁶/µL	4.5–6.5 × 10⁶/µL
Hemoglobin	13.4 g/dL	14.0–18.0 g/dL
Hematocrit	39.8%	40–52%
Mean corpuscular volume	77.4 fL	80–100 fL
Mean corpuscular hemoglobin	26.1 pg	27–34 pg
Mean corpuscular hemoglobin concentration	33.7 g/dL	32–36 g/dL
Red blood cell distribution width	15%	0–15.5%
Platelets	163 × 10³/µL	150–500 × 10³/µL
White blood cell line		
White blood cells	7.1 × 10³/µL	5.0–10.0 × 10³/µL
Neutrophils (absolute)	4.2 × 10³/µL	2.0–7.0 × 10³/µL
Lymphocytes (absolute)	2.4 × 10³/µL	0.8–4.6 × 10³/µL
Monocytes (absolute)	0.4 × 10³/µL	0.0–0.8 × 10³/µL
Eosinophils (absolute)	0 × 10³/µL	0.0–0.3 × 10³/µL
Basophils (absolute)	0.1 × 10³/µL	0–0.1 × 10³/µL
Neutrophils (%)	59.2%	50–70%
Eosinophils (%)	0.3%	1–4%
Basophils (%)	1%	0–1%
Monocytes (%)	6.3%	2–8%
Lymphocytes (%)	32.9%	25–40%
Immature granulocytes (absolute)	0.02 × 10³/µL	0–0.07 × 10³/µL
Immature granulocytes (%)	0.3%	0–0.7%
Other tests		
Erythrocyte sedimentation rate	17 mm/h	0–15 mm/h
Troponin	14.40 ng/L	<14 ng/L
pro-BNP	348 pg/mL	<125 pg/mL
Creatinine	1.1 mg/dL	0.7–1.2 mg/dL
C-reactive protein	49.60 mg/L	1.00–5.00 mg/L
Sodium	133 mmol/L	136–145 mmol/L
Potassium	4.32 mmol/L	3.50–5.10 mmol/L
Chloride	97 mEq/L	98–107 mEq/L
Blood urea nitrogen	11 mg/dL	6–20 mg/dL
Urea	24 mg/dL	17–48 mg/dL
Glucose	122 mg/dL	74–106 mg/dL
Procalcitonin	0.125 ng/mL	<0.046 ng/mL

At this time, broader antifungal coverage was available, and the patient received intravenous amphotericin B deoxycholate (1 mg/kg/day) and caspofungin (150 mg/day) in accordance with the Infectious Diseases Society of America guidelines [7] for six weeks. The patient again declined surgical intervention, citing fear of procedural risk.

Following completion of the antifungal therapy, blood cultures became negative, and the patient remained asymptomatic. He was discharged on long-term oral fluconazole suppression at a dose of 400 mg orally daily. At 12 months of follow-up, the patient remains afebrile, asymptomatic, and without clinical or microbiological evidence of relapse, completing the clinical timeline summarized in Table 2.

## Discussion

Endocarditis is a rare disease, with an annual incidence of three to seven cases per 100,000 people, yet it remains one of the most severe infectious syndromes due to its high morbidity and mortality. Approximately 25-30% of cases have been healthcare-associated, mainly related to intravascular devices or intracardiac prostheses. *Staphylococcus aureus* is the leading cause, followed by viridans group streptococci and enterococci, which together account for approximately 90% of cases [1,2].

Although uncommon (1-3%), fungal endocarditis carries a high mortality rate due to nonspecific clinical presentation and delayed diagnosis. The most frequent pathogens are *Candida* spp. (52%), *Aspergillus* spp. (24%), and *Histoplasma* spp. (7%). Major risk factors include prior cardiac surgery, immunosuppression, structural heart disease, prolonged use of antibiotics or corticosteroids, intravenous drug use, and myelodysplastic syndromes. Particular attention is warranted in patients with candidemia, prosthetic valves, or unidentified infectious sources [3,4].

Foong et al. (2020) reported a cohort of 1,873 patients with candidemia, with a 2.5% incidence of *Candida* infective endocarditis, identifying *Candida parapsilosis* as a prominent pathogen. Preexisting valvular disease was the main risk factor, and 90-day mortality reached 49%, underscoring the importance of early diagnosis and comprehensive evaluation, especially in resource-limited settings. Similarly, Lefort et al. (2012) found *Candida parapsilosis* in 16% of their fungal endocarditis cases, while Pasha et al. (2016) emphasized its predominance in patients with prosthetic valves [4,5,8].

Fungal endocarditis due to *Candida* typically arises following hematogenous dissemination, either via gastrointestinal translocation or intravascular catheters. During candidemia, *Candida* expresses adhesion factors and forms biofilms on prosthetic materials, conferring resistance to antifungal agents and immune clearance, thus promoting persistent infection [3].

Decisions on surgical intervention are complex and depend on many clinical and prognostic factors that vary among patients. The American Heart Association (AHA) recommends early valve surgery in patients with prosthetic valve endocarditis (PVE) for patients with symptoms or signs of heart failure resulting from valve dehiscence, intracardiac fistula, or severe prosthetic valve dysfunction; persistent bacteremia despite appropriate antibiotic therapy for five to seven days in whom other sites of infection have been excluded. Infective endocarditis is complicated by heart block, annular or aortic abscess, or destructive penetrating lesions, caused by fungi or highly resistant organisms. Early surgery is reasonable for patients with PVE who have recurrent emboli despite appropriate antibiotic treatment and is reasonable for patients with relapsing PVE. Early surgery may be considered in patients with mobile vegetations >10 mm [1]. The multidisciplinary team treating the patient offered surgical intervention, as they considered that the criteria present warranted this treatment; however, the patient declined on two different occasions. 

Amphotericin B remains the cornerstone of therapy for *Candida* endocarditis. Current guidelines recommend combination therapy with echinocandins or flucytosine, alongside source control through valve replacement or removal of infected devices. In a nationwide Swedish cohort, Kurland et al. (2025) analyzed 51 cases of *Candida* endocarditis treated with amphotericin B, echinocandins, or azole-based regimens, finding no significant difference in one-year mortality among groups. Echinocandins demonstrate superior in vitro activity against *Candida* biofilms, suggesting potential benefit in prosthetic-associated infections [9].

*Candida parapsilosis *shows decreased susceptibility to echinocandins as a consequence of the naturally occurring P660A amino acid substitution in the hot spot region 1 (HS1) of the FKS1 gene. Likewise, there is an increase in the number of azole-resistant isolates due to the Y132F substitution, the most common Erg11 substitution in fluconazole-resistant isolates [10].

The favorable outcome observed in this patient, despite recurrent disease, absence of surgery, and resource limitations, supports the role of prolonged combination antifungal therapy with close monitoring, supporting the role of echinocandins in *Candida parapsilosis* PVE.

## Conclusions

Fungal endocarditis caused by *Candida parapsilosis* remains a condition of high morbidity and mortality, particularly in patients with prosthetic valves and in resource-limited environments, where guideline-recommended therapies and surgical options may not be accessible. This case demonstrates that conservative medical (non-surgical) management can be a viable option under appropriate clinical conditions, consisting of combined antifungal therapy and close clinical monitoring, and can achieve favorable outcomes when surgical intervention is not feasible. It also underscores the need to tailor therapeutic strategies to local contexts and to strengthen preventive measures in patients with prosthetic valves in low-resource healthcare systems.
